# Advancement in artificial intelligence for on-farm fruit sorting and transportation

**DOI:** 10.3389/fpls.2023.1082860

**Published:** 2023-04-06

**Authors:** Zheng Zhou, Umair Zahid, Yaqoob Majeed, Nisha, Sadaf Mustafa, Muhammad Muzzammil Sajjad, Hafiz Danish Butt, Longsheng Fu

**Affiliations:** ^1^ College of Engineering, Heilongjiang Bayi Agricultural University, Daqing, China; ^2^ Department of Food Engineering, Faculty of Agricultural Engineering and Technology, University of Agriculture Faisalabad, Faisalabad, Pakistan; ^3^ College of Mechanical and Electronic Engineering, Northwest A&F University, Yangling, China

**Keywords:** deep learning, precision farming, machine vision, postharvest handling, infield transportation

## Abstract

On-farm sorting and transportation of postharvest fruit include sorting out defective products, grading them into categories based on quality, distributing them into bins, and carrying bins to field collecting stations. Advances in artificial intelligence (AI) can speed up on-farm sorting and transportation with high accuracy and robustness and significantly reduce postharvest losses. The primary objective of this literature review is to provide an overview to present a critical analysis and identify the challenges and opportunities of AI applications for on-farm sorting and transportation, with a focus on fruit. The challenges of on-farm sorting and transportation were discussed to specify the role of AI. Sensors and techniques for data acquisition were investigated to illustrate the tasks that AI models have addressed for on-farm sorting and transportation. AI models proposed in previous studies were compared to investigate the adequate approaches for on-farm sorting and transportation. Finally, the advantages and limitations of utilizing AI have been discussed, and in-depth analysis has been provided to identify future research directions. We anticipate that this survey will pave the way for further studies on the implementation of automated systems for on-farm fruit sorting and transportation.

## Introduction

**Figure 1:**
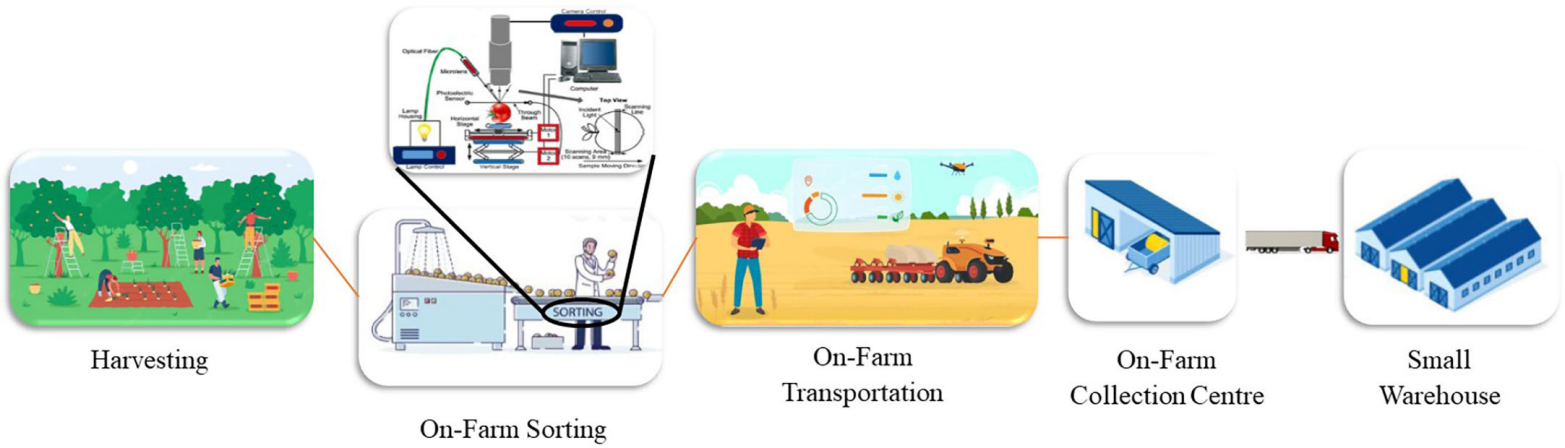
Flow diagram of operations involved in on-farm sorting and transportation.

On-farm sorting involves the removal of damaged, diseased, and rotten produce, and then categorizing the produce into bins or trays based on size, color, maturity, and ripening stage (Majeed and Waseem, 2022). The bins and trays are carried from the picking point to the collection center, which is usually at the end of a row or field. On-farm transportation is the operation of transporting bins and trays to the collection center. For large fruit-producing companies, the operations of sorting and transporting fruit for packing are conducted in the sorting line of the factory. However, in small farms that lack an integrated production line, these tasks may be conducted in the field or warehouse. On-farm sorting and transportation are important for small commercial farms and orchards because the produce is sorted and separated into bins on-site and directly delivered to storage before being sold in the fresh market. The operations of on-farm sorting and transportation are shown in **Figure 1**.

Producers are often reluctant to utilize manual on-farm sorting approaches because of higher labor costs and lower efficiency. High profits would be hard to achieve without on-farm sorting operations to remove products such as overripe, rotten, and defective fruit that are unsuitable for the fresh market. Manual on-farm transportation is a repetitive task that requires pickers to walk in the field carrying full and empty trays and bins between the picking location and the collection station. With the development of artificial intelligence (AI) technology, automated on-farm sorting and transportation have been used as an alternate and viable method to replace manual sorting and transportation of fruits. In recent years, AI-based systems have progressively been employed for on-farm sorting and transportation tasks, greatly improving the production efficiency of agriculture. The deployment of AI can speed up post-harvest handling processes and significantly reduce post-harvest losses and the chance of mechanical injury (Idama and Uguru, 2021). Additionally, it enhances the facility and safety level of human tasks for handling fresh horticultural produce (Bader and Rahimifard, 2020).

A number of AI solutions have been provided for maintaining the quality of fresh fruit products at the on-farm and post-harvest stages. In the last few years, significant reviews attempts have been devoted to the development of automated agricultural systems that are capable of efficiently performing tedious field tasks such as fruit yield estimation (Maheswari et al., 2021), shoot thinning (Majeed et al., 2020; Majeed et al., 2021), non-destructive defect detection (Nturambirwe and Opara, 2020), and mechanical harvesting (Zhang et al., 2020) by using AI applications. However, available reviews lack information on the advancement of AI techniques such as computer vision, machine learning, and deep learning for on-farm fruit sorting and transportation. Thus, a literature review is needed to determine the applications of AI for on-farm sorting and transportation of fruits, identify the challenges faced by these technologies and discuss their prospects and opportunities. On-farm sorting and transportation for small commercial farms tend to be low-cost, reliable, and miniaturized to work in conditions of machine vibration and uneven terrain, which is different from factory sorting lines and transportation. Hence, we excluded the studies on AI models for fruit quality detection for factory sorting lines and narrowed the scope to the techniques that are suitable for on-farm sorting lines and transportation, making the paper more relevant to the topic.

This paper reviews the studies on advanced technologies that have been applied to on-farm fruit sorting and transportation. Key findings related to data acquisition sensors, the use of AI models, and their benefits for on-farm sorting and transportation are presented. In addition, it addresses knowledge gaps regarding the use of AI in on-farm sorting and transportation and suggests future research directions to overcome these gaps.

## Applications of AI for on-farm sorting and transportation

### Applications of AI for on-farm sorting

Conventional fruit sorting methods are based on visual assessment. Various factors such as ripeness, quality, decay, disease, and injury are considered for sorting (Wendel et al., 2018; Gabriëls et al., 2020; Kang and Gwak, 2021). On-farm sorting requires a considerable amount of labor and low productivity is prone to human fatigue, and is biased by inspector experience, resulting in product variability and failure to meet quality standards (Rysz and Mehta, 2021). Given these challenges, recent studies have focused on the application of AI models to on-farm sorting to increase harvest automation and reduce labor costs. **Table 1** shows examples of on-farm sorting tasks solved by AI models.

**Table 1:** On-farm sorting tasks solved by AI model.

Objectives	On-farm Handling Problems	References
Defect detection	Classifying rotting and fresh fruits	Kang and Gwak (2021)
	Internal browning in mangoes	Gabriëls et al. (2020)
	Internal bruise detection in blueberries Internal defect detection in mangoes	Kuzy et al. (2018); Raghavendra et al. (2021)
Content detection	Detection of soluble solids in “Medjool” dates Chlorophyll degradation and anthocyanin detection in cherries Determination of soluble solids, starch pattern index, and Streif Index in apples Determination of soluble solids in apples	Ben-Zvi et al. (2017) Overbeck et al. (2017); Çetin et al. (2022); Wang et al. (2022)
Maturity and ripeness detection	Kiwi firmness classification	Torkashvand et al. (2017)
	Philippine coconut maturity grading	Caladcad et al. (2020)
	Firmness identification in avocadoes	Jaramillo-Acevedo et al. (2020)
	Cherry ripeness detection	Overbeck et al. (2017)
	Mango ripeness estimation	Wendel et al. (2018)
Quality (size, mass, and color) evaluation	Mass grading of mangoes	Momin et al. (2017)
	Infield grading and sorting system for apples	Zhang et al. (2021)
	Developing bin filler and on-farm sorting machine for apple harvesting	Zhang et al. (2017)

**Figure 2:**
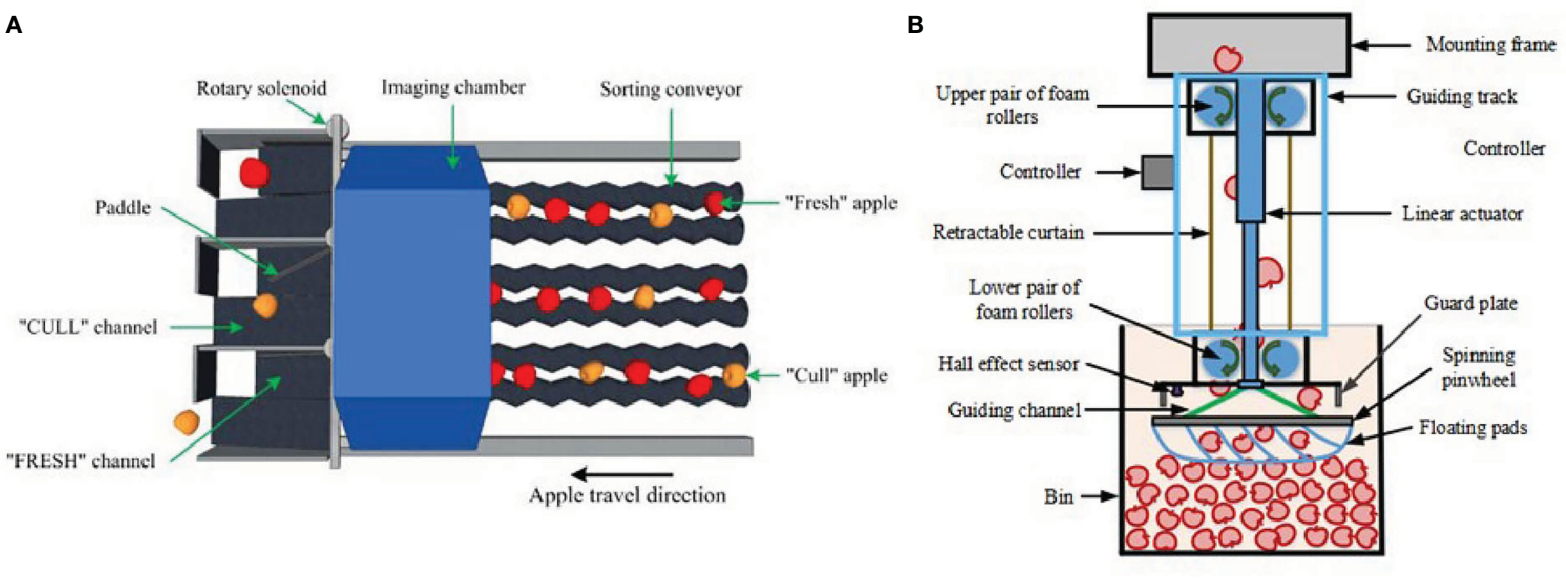
Schematic of a sorting machine: **(A)** image processing unit, fruit conveyor, and paddle; **(B)** bin filler (Lu et al., 2022).

On-farm sorting tasks that can be addressed by AI models mostly include maturity evaluation, quality identification, injury detection, and decay and disease detection for fruits such as apples, mangoes, coconuts, blueberries, etc. (Kuzy et al., 2018; Caladcad et al., 2020; Gabriëls et al., 2020; Kang and Gwak, 2021). On-farm sorting aims to remove fruit that is defective and not suitable for the fresh market and to grade fruit immediately after harvest so that it can be stored differently to achieve cost savings. AI systems have been deployed to replace human workers, overcoming visual variability, inaccuracy, and fatigue in quality grading. Machine vision is a potentially viable solution for improving harvest efficiency by providing an alternative way to automatically and non-destructively grade harvested fruits in the field. The sorting machine is highly dependent on an integrated mechanism, which is mainly composed of an image processing unit, a fruit conveyor, a paddle, and a bin filler, as shown in **Figure 2**. Implementing a computer-controlled hydraulic system for on-farm sorting machines has been greatly beneficial to achieve significant cost savings and meet commercial needs.

### Applications of AI for on-farm transportation

**Figure 3:**
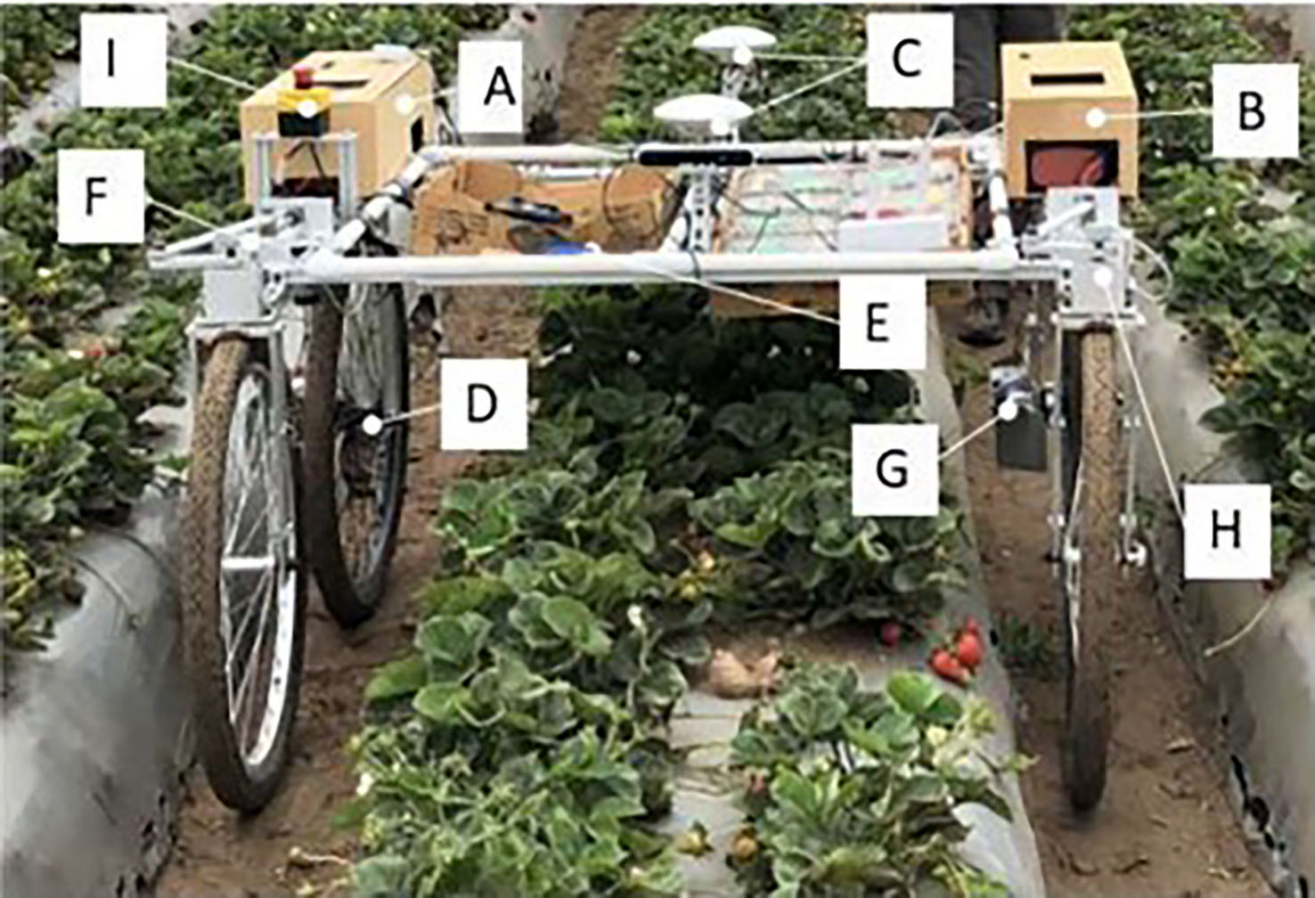
Co-robot for on-farm transportation of strawberries: **(A, B)** control boxes, **(C)** GPS antennas, **(D, G)** DC motors, **(E)** return button, **(F)** and **(H)** steer-driving system, **(I)** emergency button.

Pickers spend a considerable amount of time walking to carry full containers, i.e., trays and bags, to the collection center at the end of the row and walking back with the empty container to resume picking. Workers who use the conventional manual picking method are at risk of musculoskeletal problems due to carrying heavy loads and repetitive hand and upper body activities. Faced with the challenges of labor costs, low harvesting efficiency, and occupational injuries, harvesting aids such as on-farm transportation co-robots (**Figure 3**) have been introduced to assist in harvesting fruits such as apples, strawberries, and grapes (Ye et al., 2018; Faheem et al., 2021; Peng et al., 2021; Lu et al., 2022).

Previous research on on-farm transportation has focused on co-robot scheduling, maneuvering strategies, and minimizing damage to products during transportation. With the goal of developing a co-robot to carry trays and transport fruit in the harvest field, human-robot collaboration models that include picking and walking have been investigated for co-robot scheduling and dispatching policies (Seyyedhasani et al., 2020; Peng et al., 2021; Rysz and Mehta, 2021). Faced with the challenges of bin handling in commercial orchards, navigation, and maneuvering strategies for bin carriers have been proposed to achieve autonomous navigation between tree rows to perform bin handling tasks (Ye et al., 2018; Blok et al., 2019). A fleet of robotic N-trailer vehicles has also been introduced to assist with crop transportation as an alternative to the ultimate mechanization of manual harvesting activities to improve field efficiency (Guevara et al., 2021). To minimize the mechanical damage induced by vibration during post-harvest handling, the vibration mechanism of hanging grape clusters was analyzed to achieve low vibration of the fruit during on-farm transportation (Faheem et al., 2021).

## Data acquisition sensors and techniques for on-farm sorting and transportation

### Data acquisition sensors and techniques for on-farm sorting

Multiple sensors have been used for image acquisition in on-farm sorting and transportation, including a red-green-blue (RGB) camera, a charge-coupled device (CCD) camera, a hyperspectral camera, a near-infrared (NIR) sensor, visible and near-infrared spectroscopy, and a thermal camera. According to previous studies, the RGB camera is currently the most widely employed for on-farm sorting, especially for surface damage detection, color grading, mass and volume estimation of apples, and ripeness of avocados (Jaramillo-Acevedo et al., 2020; Lu et al., 2022; Mansuri et al., 2022). A CCD camera was utilized for the size and color grading of apples and mass grading of mangoes (Momin et al., 2017; Zhang et al., 2021). A thermographic imaging system combining a thermal camera (7.5–13 µm) with heat lamps was built for internal bruise detection in blueberries (Kuzy et al., 2018). A hyperspectral camera mounted on a ground-based mobile platform was used to detect ripeness in mango and apples (Wendel et al., 2018; Çetin et al., 2022; Wang et al., 2022). A NIR spectrometer (850–1,888 nm) was used for spectral measurements to determine the total soluble solid content in “Medjool” dates to predict fruit quality (Ben-Zvi et al., 2017). VNIR spectroscopy (200–1,000 nm and 673–1,100 nm) was employed for internal browning in mangoes (Gabriëls et al., 2020; Raghavendra et al., 2021). In addition to imaging and spectral techniques, a tapping system using an omnidirectional microphone to record the tapping sound was developed for ripeness detection in coconuts (Caladcad et al., 2020). **Table 2** represents the key instruments required for data acquisition for on-farm fruit sorting.

**Table 2:** Key instruments required for on-farm sorting data acquisition.

Key Instruments	Cultivar and application	References
RGB camera and depth camera	Freshness classification of apples, bananas, oranges, lemons, pears, and strawberries Mass and volume detection in apples Ripeness detection in avocadoes Rot detection in strawberries	Kang and Gwak (2021); Mansuri et al. (2022); Jaramillo-Acevedo et al. (2020); Lu et al. (2017)
CCD camera	Mass determination in mangoes Size and color grading in apples	Momin et al. (2017); Zhang et al. (2021)
(Visible) NIR spectrophotometer	Determination of the total soluble solids content in “Medjool” dates Internal browning measurement in mangoes Internal defect detection in mangoes	Ben-Zvi et al. (2017); Gabriela et al. (2020); Raghavendra et al. (2021)
Thermal camera	Bruise detection in blueberries	Kuzy et al. (2018)
Hyperspectral camera	Ripeness estimation of mangoes Determination of soluble solids content, starch pattern index, and Streif Index in apples Determination of soluble solids content and firmness in apples	Wendel et al. (2018); Wang et al. (2022); Wang et al., (2021)
Penetrometer and refractometer (to measure dissolved solids and firmness)	Kiwi firmness detection	Torkashvand et al. (2017)
Microphone (to record acoustic signals)	Ripeness detection in coconuts	Caladcad et al. (2020)

The RGB camera is the most employed sensor for automated on-farm sorting and grading. It should be noted that using a machine vision-based AI algorithm to sort and grade fruits and vegetables need high-quality and decipherable images. Hence, an RGB camera is the most appropriate sensor for the job. RGB cameras are low-cost and can be used to measure texture, color, geometric shape, and defects in fruits and vegetables. The main disadvantage of an RGB camera is its sensitivity to changes in illumination under different lighting conditions. Besides RGB cameras, CCD cameras are also widely used for on-farm sorting because of their low cost. Hyperspectral, NIR, and thermal cameras are more expensive sensors and provide different spectral ranges from visible light, which are universally utilized for internal defect and ingredient detection due to their sensitive spectral range for internal damage and composition. Hyperspectral imaging includes a large amount of spectral data, providing more information for defect and contaminant detection in on-farm sorting. However, the cost is the major drawback of hyperspectral cameras.

### Data acquisition sensors and techniques for on-farm transportation

With regards to cameras and sensors employed for on-farm transportation, LiDAR sensors, and positioning systems are more commonly used than vision-based sensors due to their robustness for robot localization and navigation in outdoor environments. A 2D LiDAR scanner was placed on the front of the orchard transport robot to observe tree growth on each side (Blok et al., 2019). Positioning systems such as GNSS, GPS, RTK-GNSS, and RTK-Global Positioning System (RTK-GPS) were used to obtain ground truth position information and schedule the path for the bin-handling machines (Ye et al., 2018; Blok et al., 2019; Peng and Vougioukas, 2020; Peng et al., 2021). An inertial measurement unit (IMU) was employed for the measurement of the location and heading angle of on-farm harvesting-aid robots (Ye et al., 2018; Blok et al., 2019). A wheel encoder was used to measure wheel odometry (Blok et al., 2019). A load cell was used to sense the load on the tray to calculate the amount of fruit collected for transportation path scheduling (Anjom and Vougioukas, 2019; Peng and Vougioukas, 2020; Peng et al., 2021). A uniaxial force sensor was inserted between the grape clusters to calculate the peak hanging force and realize the vibrations generated in the grape clusters during postharvest handling (Faheem et al., 2021). **Table 3** shows the key instruments required for data acquisition during on-farm transportation.

**Table 3:** Key instruments required for on-farm transportation data acquisition.

Key Instruments	Cultivar and application	References
Force sensor	Hanging force analysis for grape clusters during on-farm transportation	Faheem et al. (2021)
Positioning system	Navigation system for bin transportation in the orchard Navigation system for bin transportation in the orchard Navigation system for tray transportation in strawberry fields	Ye et al. (2018); Blok et al. (2019); Peng and Vougioukas (2020); Peng et al. (2021)
LiDAR	Obstacle detection for bin-handling robot in the orchard Fruit detection of apples Tree segmentation	Blok et al. (2019); Geno-Mola et al. (2019); Wendel et al. (2018)

In previous studies, LiDAR sensors have been most commonly used for on-farm transportation. They have been widely used for yield estimation and geometric measurements of canopy value at the harvest stage due to their ability to be unaffected by illumination (Westling et al., 2020; Chen et al., 2022; Yu et al., 2022). For on-farm transportation, the main ability of LiDAR is that it can directly deliver 3D localization data to co-robots navigating in the field and orchard. Positioning systems such as GNSS, GPS, RTK-GNSS, and RTK-GPS have been employed to obtain the locations of co-robots for bin and tray scheduling. Other sensors, such as the IMU, wheel encoder, load cell, and force sensor, have been adopted to control on-farm vehicles and quantify harvesting efficiency for co-robot route planning. The accuracy of the sensors is crucial to the localization and navigation of on-farm transport vehicles. With the development of sensor technology, the accuracy of LIDAR scanners and positioning systems has been limited to the centimeter level, which is within the tolerance of precision agriculture (Ye et al., 2018; Blok et al., 2019). The accuracy of the IMU used to measure the heading angle of the on-farm vehicle was 0.1–5°. The accuracy and details of other sensors, such as wheel encoders, load cells, and force sensors, employed to evaluate harvest efficiency were not mentioned in the relevant literature, but they can be considered suitable for on-farm vehicles based on the efficiency of the experimental results.

## AI models for on-farm sorting and transportation

### AI models for on-farm sorting

AI models have been used for on-farm sorting along with the significant development of machine learning, deep learning, and machine vision techniques. On-farm grading is mostly aimed at identifying the size, mass, ripeness, surface and internal defects, and diseases in fruit for further transportation, storage, and packaging. Surface and internal defect detection for fruit classification is useful to improve product quality and boost profits. Moreover, on-farm defect detection could analyze product degradation and damage and take preventative actions to halt the deterioration process. Fruit firmness analysis can assist in improving the packaging process by maintaining consistency. AI-based techniques provide a solution for automatically unloading graded fruit into bins to improve harvesting efficiency.

Various image processing methods and deep learning models have been introduced to achieve automated on-farm sorting. Artificial neural network (ANN) and support vector machine (SVM) models have been developed to estimate the mass and volume of apples and mangoes (Utai et al., 2019; Mansuri et al., 2022). Classification of bruises in blueberries was performed using linear discriminant analysis, SVM, random forest (RF), K-nearest-neighbors, and logistic regression classifiers (Kuzy et al., 2018). ANN, RF, SVM, convolutional neural network (CNN), multiple linear regression (MLR), and partial least squares (PLS) were used to classify the ripeness and firmness levels of apple, coconut, kiwi, and mango (Torkashvand et al., 2017; Torkashvand et al., 2017; Wendel et al., 2018; Caladcad et al., 2020; Wang et al., 2022). ANN and PLS were used for identifying browning and total soluble solids in “Medjool” dates and mangoes for quality measurement (Ben-Zvi et al., 2017; Gabriëls et al., 2020). The performance of the AI models is presented in **Table 4**.

**Table 4:** Accuracy of the AI models for on-farm handling.

AI Models	Objectives	Best Accuracy Achieved	References
ResNet-50, ResNet-101	Freshness classification for apples, bananas, oranges, lemons, pears, and strawberries	98.50%	Kang and Gwak (2021)
PLS	Detection of soluble solid content in “Medjool” dates	RMSE = 0.9	Ben-Zvi et al. (2017)
ANN	Kiwi firmness detection	RMSE = 0.539 R^2^ = 0.724	Torkashvand et al. (2017)
ANN, RF, SVM,	Determination of ripeness level in coconuts	ANN of premature coconut = 38% RF of premature coconut = 25% SVM of premature coconut = 38% ANN of mature coconut = 44% RF of mature coconut = 59% SVM of mature coconut = 38%	Caladcad et al. (2020)
ANN ANN	Internal browning measurement in mangoes Determination of soluble solid content and firmness in apples	Over 80% R^2^ = 0.724 for firmness detection R^2^ = 0.724 for soluble solid content detection	Gabriëls et al. (2020) Çetin et al., 2022
SVM, RF, LAD, KNN, logistic regression	Bruise detection in blueberries	Accuracy (Farthing cultivars with logistic regression) = 90% Accuracy (Meadowlark cultivars with RF) = 80%	Kuzy et al. (2018)
ANN	Ripeness classification in avocadoes	Classification accuracy = 88%	Jaramillo-Acevedo et al. (2020)
SVM	Apple size and volume estimation	R^2^ = 0.955 for size estimation R^2^ = 0.965 for volume estimation	Mansuri et al. (2022)
ANN	Mass grading of mangoes	Accuracy (diameter) =97% Accuracy (perimeter) = 79% Accuracy (roundness) = 36%	Utai et al. (2019)
CNN, PLS	Ripeness estimation of mangoes	RMSE (CNN) = 1.08% RMSE (PLS) = 1.17% F1 (CNNandPLS) >0.97	Wendel et al. (2018)

Spectroscopy techniques such as hyperspectral spectroscopy and visible and near-infrared spectroscopy, which include large amounts of data, have been used for the chemical detection of various fruits due to their sensitivity to nutritional and constituent content that can be detected by external inspection (Ben-Zvi et al., 2017; Jaramillo-Acevedo et al., 2020; Çetin et al., 2022). ANN models, which are robust for pattern recognition in a large amount of data, are used for hyperspectral imaging analysis in chemical detection, such as total soluble solids content, mineral nutrient content, and dry matter content for mangoes, bananas, blueberries, etc. Visible image size and external defects. ANN models have shown great potential in grading, which are superior to other models such as PLS, SVM, and RF (Wendel et al., 2018; Gabriëls et al., 2020). Visible imaging has been employed to determine physical features such as size, mass, color, and external defects and bruises in fruits. CNN models have been introduced to replace manual inspection in fruit sorting by extracting physical features from images and using them to detect the size, mass, color, and external defects and bruises (Wendel et al., 2018; Kang and Gwak, 2021). ANN and CNN models provide solutions for making quality decisions and improving postharvest efficiency through on-farm sorting, which will be soon widely adopted for automated on-farm sorting systems, especially in small commercial farms and orchards.

### AI models for on-farm transportation

Workers’ picking speeds vary based on fruit distribution and the individual’s work pace. To address this issue, crop transport harvesting-aid robots have been developed to manage full and empty bins/trays as harvesting aids to increase the productivity, safety, and aesthetics of manual harvesting (Fei and Vougioukas, 2021; Guevara et al., 2021). Since harvesting-aid robots are subject to the constraints of space between crop rows and the cooperation with pickers, maneuvering strategies are required to determine the transport path and operation to deliver full and empty bins in the field. Modeling frameworks for the combined tasks of manual harvesting and robot-assisted crop transport have been proposed to support specialty crops that require labor to distribute and pick fruit. Models describing the interactions between robots and humans during fruit harvesting, such as deterministic predictive dynamic scheduling (Peng and Vougioukas, 2020), a stochastic model (Seyyedhasani et al., 2020), and a risk-averse optimization approach (Rysz and Mehta, 2021), have been utilized to identify optimal harvester servicing policies. Evaluating the efficiency of pickers is vital for scheduling co-robots to transport empty and full bins in the field. Mechanistic gray box models were addressed to predict the time when a picker will fill up the tray being currently harvested to calculate tray transport request time, with the goal of increasing harvesting efficiency (Anjom and Vougioukas, 2019). Field navigation is another core feature for on-farm transport harvesting-aid robots, which includes co-robot localization, a vehicle steering scheme, and path scheduling. Probabilistic localization algorithms, i.e., the Kalman filter and the particle filter using a 2D light detection and ranging (LiDAR) scanner, were developed for in-row robot navigation in orchards (Blok et al., 2019). Maneuvering strategies were created for four steering vehicles to complete different bin-handling tasks in commercial orchards (Ye et al., 2018). To support cooperation between pickers and harvesting-aid robots, cooperative navigation strategies were used to generate a feasible path to arrange and allocate vehicles and trailers for transporting empty and full trays between field rows (Guevara et al., 2021; Peng et al., 2021).

Small crews of harvesting-aid robots that assist big picking crews by transferring full and empty trays can boost harvesting efficiency by minimizing pickers’ unproductive walking times. The study of robotic postharvest handling can benefit specialty fruits such as blueberries, lychees, grapes, and cherries. Harvesting-aid robots have been used in conjunction with pickers, following the departure times, harvesting sequence, computed vehicle routes, and the number of trailers as guidelines. Harvesting-aid robots can handle the transport of empty and full bins and trays between the collection stations and the pickers, thus improving harvesting efficiency by decreasing the unproductive walking times of the pickers in the field. A good maneuvering strategy for a four-wheel independently steered robotic bin-handling machine can effectively accomplish the navigation of bin transport on the desired trajectories without damaging fruit trees or hitting bins. Hanging force analysis for grape clusters during on-farm transportation is helpful to realize the low vibration of fruit clusters, which provides the theoretical basis for reducing berry drop during on-farm transportation (Faheem et al., 2021).

## Challenges and future directions for AI in on-farm sorting and transportation

### Challenges and future directions for AI models in on-farm sorting

I. The most common sensors used for on-farm sorting are RGB and CCD cameras. However, RGB and CCD cameras can only detect surface and superficial appearance parameters such as shape, color, and size. There is a need for sensors that meet the requirements for high throughput function and low cost before this technology is more widely adopted for small farms and orchards. Automated sorting and grading systems using sensors such as hyperspectral cameras, lasers, and NIR for defect detection have been developed for factory sorting lines. To improve the performance of internal disease and defect detection in the factory line, more advanced sensors, and techniques could be adopted and modified to identify surface and internal diseases and defects of fruits and vegetables for the on-farm sorting system.
II. AI models have been more universally developed to determine maturity level by fruit shape, size, mass, and nutritional substance for harvesting than for in-field sorting and grading. Accordingly, AI models for harvesting have achieved better performance than the ones for on-farm sorting. To improve the performance of on-farm sorting to address this problem, research such as that on ripeness and quality detection (Overbeck et al., 2017; Halstead et al., 2018) for fruit and vegetable harvesting could be introduced and modified. Furthermore, AI models and systems for on-farm sorting need to be further optimized based on the factory sorting line to meet the requirements of working in field conditions, which requires systems that are reliable and prone to being functional to work with machine vibrations and uneven terrain with a high sorting throughput.
III. Another limitation is the dataset used for the AI model of on-farm sorting. In previous studies, datasets were mostly built for individual research using limited varieties and cultivars. Fruit samples were randomly selected to ensure the diversity of the dataset, and the fruit quality used as a reference for the AI models was manually checked and classified by experts (Caladcad et al., 2020; Mansuri et al., 2022; Wang et al., 2022). These data collections to build datasets were conducted for individual research, which could lead to tendencies of model overfitting and reduce the accuracy of the AI model. Additionally, AI models may be biased as data collection was conducted either indoors or outside, with varying environmental conditions affecting illumination. As a result, the diversity of datasets for on-farm sorting has not been fully developed. Hence, it is suggested that we use additional data samples, including more diverse production circumstances and different cultivars, to improve the generalization ability of AI models. Open-source image datasets could be another solution for expanding the samples to avoid overfitting in AI model training. Furthermore, a findable and accessible dataset that includes more fruit types and varieties conducted in different environmental conditions would be beneficial to expand the dataset for generalization and development of AI algorithms and to prompt the on-farm sorting industry for small farms and orchards.
IV. On-farm post-harvest processing of fruit comprises a series of operations, including sorting and grading, bin filling, cleaning, and waxing. Most of the studies have focused on the on-farm processing of sorting, grading, and bin filling. There are few studies related to the on-farm cleaning and waxing of fruit. Small farms and orchards usually skip the process of cleaning and waxing and deliver the produce directly to the market due to the lack of low-cost cleaning and waxing machinery. Without cleaning and waxing, the shelf life of the fruit would be greatly compromised during storage, which would ultimately affect the quality and profit of the fruit. Low-cost on-farm cleaning and waxing machinery could be a solution for small farms and orchards. The development of on-farm cleaning and waxing machinery integrated with sorting and grading systems can be an opportunity to improve the robustness and efficiency of on-farm handling on small farms and orchards.
V. Studies of on-farm handling of vegetables are rarely found in the published literature. This is because most vegetables are less profitable than fruits, so farms would rather invest less in AI and robotics for the on-farm sorting of vegetables to balance inputs and outputs. However, organic vegetables, which are more dependent on skilled management, have become popular in the fresh market, providing an opportunity for the development of AI in the on-farm sorting of specialty vegetables such as tomatoes, peppers, and cucumbers. We anticipate that there will be more demand for AI applications in the on-farm sorting of vegetables in the near future.
VI. Initially, researchers only focused on the AI models for the sorting lines of big commercial producers, and the AI models tended to be developed for sorting the produce that was directly transported from farms and orchards to factories, rather than for the on-farm stage that occurs before transportation (Nasiri et al., 2019; Yang et al., 2022). Small farms and orchards had limited access to automated fruit sorting lines due to their high cost. The development of sensor technology and AI models has opened up the potential for automated fruit sorting in small farms and orchards. In recent years, customer demand for high-quality produce has promoted the on-farm management of small farms and orchards. Consequently, small producers have gradually realized the importance of replacing costly labor with automated sorting systems to save costs and improve efficiency, whereupon AI models for on-farm sorting systems have been introduced. However, AI models for on-farm sorting are still less focused on researchers compared to those for big factories. The limitation of factory sorting used for on-farm sorting is that the AI models and sensors may not be efficient for the on-farm environment due to operational demands such as lighting, working conditions, and throughput requirements. As a matter of fact, the purpose of factory and on-farm sorting is the same: to sort and grade produce and remove unwanted items. Therefore, an on-farm fruit sorting system could adopt the AI models developed for factory sorting lines due to their prevalence and effectiveness, but it should be modified and fine-tuned to meet the requirements and environmental conditions of an on-farm operation. Meanwhile, the development of flexible and low-cost sorting lines for on-farm sorting could provide solutions for reducing the costs for big factories. Hence, it is anticipated that the development of AI models for fruit sorting would be beneficial for both factory and on-farm sorting.

### Challenges and future directions for AI models in on-farm transportation

I. Harvesting-aid robots have been introduced to transport empty and full trays between picking crews and the collection station at the end of the field. Collaboration models between pickers and robots have been studied for harvesting-aid robot scheduling to increase harvest efficiency. Reactive scheduling has been replaced by predictive scheduling because it can reduce or eliminate the wait time for the robot to move to the pickers’ location. Using co-robot scheduling models, harvesting-aid robots for fragile fruits such as strawberries and table grapes have been introduced as prototypes and simulated using manual harvest data. However, most of the on-farm transporting harvesting-aid robots have not been practically applied in commercial farms or orchards. Mechanizing harvesting-aid robots, which can be practically used for transporting empty and full trays in commercial fields, constitutes one of the biggest challenges for on-farm transportation. Harvesting-aid robots integrate technologies such as automatic navigation, localization, wireless data transmission, and maneuvering strategies. Further work will involve combining these technologies to improve the practicality of harvesting-aid robots. The core part of on-farm harvesting-aid robots is route planning. Advanced computing technologies such as search algorithms have been developed to meet the requirements of the logistics industry. The search algorithm can find the best solutions for the desired goal by searching for all the possible alternatives, which is achieved in two phases, i.e., problem definition and search space exploration. The search algorithm has shown its efficiency and accuracy in multi-robot navigation and cooperation (Xu et al., 2020; Sahu et al., 2023). AI models based on search algorithms can potentially be used for on-farm harvesting-aid robots to minimize the energy cost and time taken in complex on-farm tasks.
II. Safety and collision issues are another concern for on-farm transport as most transport robots adopt route planning strategies for navigation. Obstacle and headland detection are crucial for orchard robots to avoid endangering humans and obstacles on the navigation path. In addition, they need to turn or stop when encountering extreme terrain and at the edge of the road. However, this problem has not been previously addressed and needs to be further investigated for on-farm transport robots. Although positioning systems such as RTK-GNSS have been utilized to identify environmental scenarios in farming vehicles, the tree canopies close to the vehicles can obstruct the satellite signals and degrade the accuracy. This can be addressed by better-utilizing sensors such as 3D LiDAR and RGB-depth cameras and integrating them with AI models to classify objects, areas, and paved and unpaved roads in orchards. The implementation of automatic driving and obstacle avoidance for on-farm transport could improve human safety and prevent collisions between robots and vehicles. Promisingly, on-farm transport robots integrated with positioning systems and machine vision systems for inter-vehicle coordination and safe navigation will be the focus of research in the near future.
III. Mechanical damage induced by the vibration of produce during bin filling and on-farm transportation can reduce the quality and shelf life of fruits and vegetables. Bruising and abrasions can be exacerbated by overweight bins and bumps in the uneven terrain of fields and orchards. Only a few studies have focused on bin management to overcome mechanical damage during bin filling and on-farm transport. On-farm bin fillers, which are usually incorporated with on-farm sorting and grading platforms, play an important role in evenly distributing fruits and vegetables to reduce bruising and abrasion. Sensors such as an IR sensor and a Hall-effect sensor have been used for bin fillers to measure the fall distance between the fruit and the filler for bruise prevention during catching and distributing the fruit into bins (Peng et al., 2022). However, it can only detect the distance between the filler and the area directly below the sensor, resulting in inaccuracy in the distance and distribution evaluation of fruits and vegetables. This problem can be addressed by introducing AI techniques using sensors such as a depth camera or 3D LiDAR, which provide a wider field of view of the fruit load distribution and thus better control the position of the bin filler, preventing mechanical damage during bin filling.
IV. Previous studies have focused on on-farm bin handling from the perspective of maneuvering strategies and vehicle positioning. The decision-making process for storage and transportation at the post-harvest stage plays a crucial role in the long-term stability of produce. To achieve the goal of planning post-harvest storage and transportation, farmers, and distributors need to monitor the on-farm harvesting process. Bin monitoring is one of the main ways to monitor the harvesting process, but it has rarely been studied in the literature. Load cells have been used to measure the number of trays to track the picking speed of strawberries for transport co-robot arrangements, which could alternatively be applied to measure the quantity of bin weight to determine the harvesting speed. Furthermore, smart bins equipped with sensors that can detect the condition of fruits and vegetables inside the bins, such as temperature, humidity, ethylene, and carbon dioxide production, can be a powerful tool to make appropriate decisions for storage and transportation at the post-harvest stage.

## Conclusion

Advanced technologies in AI are increasingly being used to automate a variety of on-farm sorting and transportation operations. In recent years, AI approaches have proven to be useful and effective for automated fruit sorting and transportation at the on-farm post-harvest stage. This paper focused on determining the applications of AI for on-farm fruit sorting and transportation, identifying the challenges faced by these applications, and discussing the prospects and opportunities. Key findings related to data acquisition sensors, the use of AI models, and their merits are presented. Limitations and future research directions have been elaborated to provide an overview and in-depth analysis of potential applications of AI for on-farm sorting and transportation. We believe that these investigations will help direct future research toward building autonomous systems for on-farm sorting and transportation. In the context of increasing labor costs and the demand for better-quality produce in the fresh market, there will be a great need for AI-based systems for on-farm sorting and transportation. Given the rapid advancement of AI technologies, it is predictable that the use of AI for on-farm sorting and transportation will have a widespread application in precision agriculture in the near future.

## Author contributions

ZZ and UZ contributed in editing of draft of manuscript. YM contributed in conceptualization, project administration, and editing of final draft of manuscript. N, SM, MMS, and HDB contributed in survey of literature, graph preparation. LF contributed in evaluation of manuscript. All authors contributed to the article and approved the submitted version.
